# Diversity in the Extracellular Vesicle-Derived Microbiome of Tissues According to Tumor Progression in Pancreatic Cancer

**DOI:** 10.3390/cancers12092346

**Published:** 2020-08-19

**Authors:** Jin-Yong Jeong, Tae-Bum Kim, Jinju Kim, Hwi Wan Choi, Eo Jin Kim, Hyun Ju Yoo, Song Lee, Hye Ryeong Jun, Wonbeak Yoo, Seokho Kim, Song Cheol Kim, Eunsung Jun

**Affiliations:** 1 Department of Convergence Medicine, Asan Institute for Life Sciences, University of Ulsan College of Medicine and Asan Medical Center, Seoul 05505, Korea; jyjeong@amc.seoul.kr (J.-Y.J.); 83pearl@hanmail.net (J.K.); chlgnldhks@gmail.com (H.W.C.); kokoejko@gmail.com (E.J.K.); yoohyunju@amc.seoul.kr (H.J.Y.); 2 Department of Allergy and Clinical Immunology, Asan Medical Center, University of Ulsan College of Medicine, Seoul 05505, Korea; tbkim@amc.seoul.kr; 3 Division of Hepatobiliary and Pancreatic Surgery, Department of Surgery, Asan Medical Center, University of Ulsan College of Medicine, Seoul 05505, Korea; ssong277@hanmail.net (S.L.); lovelyhye9@gmail.com (H.R.J.); 4 Environmental Disease Research Center, Korea Research Institute of Bioscience and Biotechnology, Daejeon 34141, Korea; aureus79@gmail.com; 5 Department of Medicinal Biotechnology, College of Health Sciences, Dong-A University, Busan 49315, Korea; cvaccine@dau.ac.kr; 6 Biomedical Engineering Research Center, Asan Institute of Life Science, AMIST, Asan Medical Center, Seoul 05505, Korea

**Keywords:** microbiome, extracellular vesicle, tissue, tumor progression, pancreatic cancer

## Abstract

This study was conducted to identify the composition and diversity of the microbiome in tissues of pancreatic cancer and to determine its role. First, extracellular vesicles (EVs) were obtained from the paired tumor and normal tissues, and 16s rRNA gene sequencing was performed. We identified the microbiomes, compared the diversity between groups, and found that *Tepidimonas* was more abundant in tumors. Second, larger tumors resulted in lower levels of *Leuconostoc* and *Sutterella*, and increased lymph node metastasis resulted in higher levels of *Comamonas* and *Turicibacter* in tumor tissues. Moreover, in the case of tumor recurrence, the levels of *Streptococcus* and *Akkermansia* were decreased in tumor tissues. Finally, with the supernatant of *Tepidimonas* *fonticaldi*, proliferation and migration of cells increased, and epithelial-mesenchymal transition and the Tricarboxylic Acid (TCA) cycle-related metabolites were enhanced. The composition and diversity of EV-derived microbiomes are important for providing novel insights into theragnostic approaches in pancreatic cancer.

## 1. Introduction

The 5-year survival rate of patients with pancreatic cancer is less than 10% [1], and it is predicted to be the second most common cause of cancer-related deaths by 2030 [2]. Despite advances in diagnostic technology for blood analysis and imaging modalities, only 20–30% of patients with pancreatic cancer can undergo curative resection. Moreover, only some of these patients have a survival rate of over 5 years [1,3]. In order to increase the vicious survival rate, various studies have been conducted to improve the rate of early diagnosis and to establish a molecular-genomic landscape related to long-term survival [4,5]. Interestingly, it has recently been reported that the diversity and composition of oral or gut microbiome are also associated with oncogenesis, immune response, and patient survival in pancreatic cancer [6,7,8].

From the beginning of the 19th century, when infectious diseases played an important role in determining the mortality of patients, studies of microbiota (including bacteria, virus, fungi, and protozoa) have been actively conducted, and these studies have been extended to various areas of cancer research including carcinogenesis [6]. The microbiome is a combination of microbiota and their collective genome, and this area has been widely studied with the development of next-generation sequencing techniques [9,10]. Representatively, oral and gut microbiomes form a unique metagenome, which are dynamically changing according to nutrition, geography, sex, age, etc. They are known to play a vital role in nutrition digestion, immune response, and carcinogen metabolism [11,12]. Consequently, dysbiosis (a corrupted and imbalanced status of the microbiome) has been reported to be associated with the development and progression of various diseases [13]. In particular, dysbiosis of the gut microbiome and disruption of the epithelial barrier can induce microbiota translocation, which is thought to alter several neoplastic transformations and to alter the tumor microenvironment [7,14]. The microbiome has been reported to play an important role in oncogenesis and the immune microenvironment of gastric, colon, and liver cancer, which are direct contacted with gut microbiome or received blood from the portal vein (PV) of the gastrointestinal tract [15,16,17].

Studies of *Helicobacter pylori* (*H. pylori*) in pancreatic cancer have been performed for decades; however, the results of its association with patient survival and its role as a risk factor are not consistent and remain controversial [18,19]. Nevertheless, the importance of microbiomes in pancreatic cancer has been reported in recent studies. Oncogenesis can be promoted by controlling the innate and adaptive immune system [7]. Furthermore, the response to cancer immunotherapy can be controlled [6,20], and the composition and diversity of microbiomes can affect patient survival [8]. Despite these findings, studies of the microbiome are limited in pancreatic cancer compared with other tumor types; thus, it is necessary to investigate the effects of microbiomes at the various stages of pancreatic cancer. In particular, considering that the pancreas is located in a position that is not directly in contact with the gut microbiome, studies of the various functional units of microbiota such as microbes, metabolites, genes, and proteins are needed [21].

We hypothesized that the composition and diversity of microbiomes in tumor and normal tissues of patients with pancreatic cancer would be different and that these differences would have distinctive effects on tumor development. To investigate this, we obtained extracellular vesicles (EVs) from normal and tumor tissues of patients with pancreatic cancer and performed a microbiome analysis. We also attempted to determine whether these differences depend on tumor progression and tumor recurrence and whether the microbiota could regulate the oncological function of tumor cells.

## 2. Results

### 2.1. Patient Characteristics

In this study, the tumor and normal tissues from 15 patients diagnosed with pancreatic ductal adenocarcinoma (PDAC) were analyzed (Table 1 and Table S1). Albumin (Alb), blood urea nitrogen (BUN), and creatinine (Crea) were within the normal range, and carbohydrate antigen 19–9 (CA 19–9) was increased to 190.3 U/mL. Pathological examination revealed that the location of the tumor was dominant (66.7%) on the body/tail side; thus, the operation tended to be distal pancreatectomy and splenectomy (66.7%). The average tumor size was 3.3 cm, and more patients had metastatic lymph nodes (66.7%). One patient (patient no. 12) underwent chemotherapy before surgery, and 8 patients (53.3%) had tumor recurrence within 4 years.

### 2.2. Tissue-Specific EV-Derived Microbiomes

**Figure 1:**
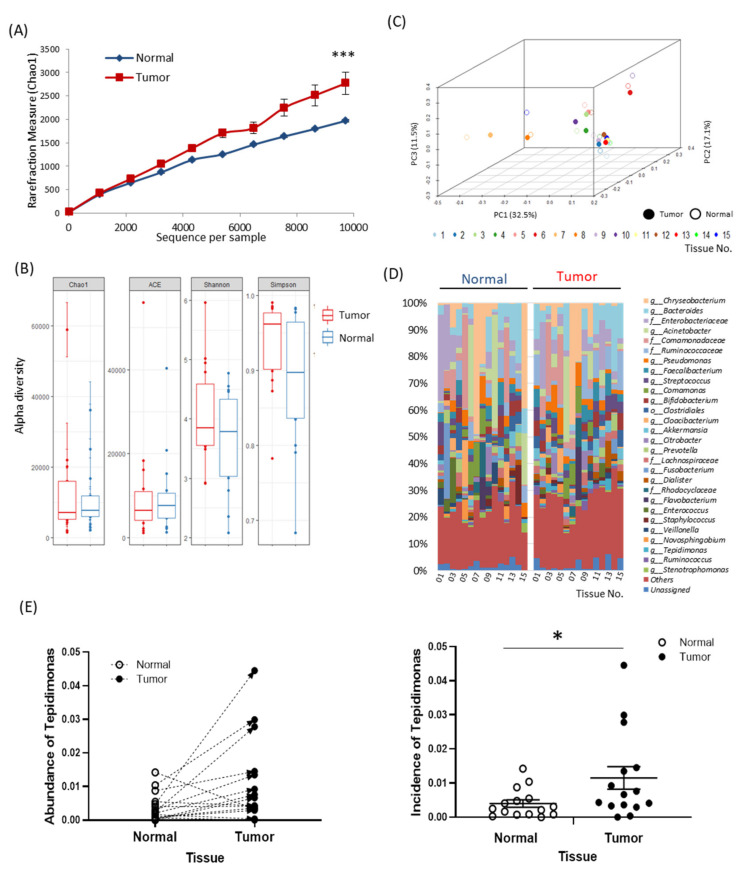
Characterization of extracellular vesicles (EVs) in the tissues of patients with pancreatic cancer and microbiome composition profiling: (**A**) alpha diversity rarefaction plots for the EV-derived microbiomes in tumor and normal tissues (*n* = 15, mean ± SEM, *** *p* < 0.001), (**B**) comparison of alpha diversity indices between tumor and normal tissues (*n* = 15), (**C**) beta diversity of EV-derived microbiomes in 3-dimension in tumor and normal tissues (*n* = 15), (**D**) common microbiota in paired normal and tumor tissues at the genus level (*n* = 15), and (**E**) abundance of Tepidimonas in tumor and normal tissues (*n* = 15, mean ± SEM, * *p* < 0.005).

EVs were obtained from all 15 paired normal and tumor tissues, and the average size of the EVs was 63.42 nm (Figure S1). The microbiomes of EVs with sequences per sample were compared, and species richness in the tumor tissues was increased significantly with increasing sequences (Figure 1A, *p* < 0.001). A comparison of the diversity of the whole microbiome between groups revealed that there was no significant difference in the Chao1, ACE, Shannon, and Simpson methods (Figure 1B). The compositions of the microbiota for each individual from the phylum to genus levels are shown using the 2-dimensional (Figure S2) and 3-dimensional (Figure S3 and Figure 1C) techniques. Based on the microbiota frequencies in each taxonomic rank, the compositions in tumor and normal tissues are expressed as a heat map (Figure S4 and Figure 1D) and hierarchical clustering (Figure S5) was performed. In order to confirm whether the gut microbiota can translocate to tissue EVs, the compositions of *Bacteroides*, *Blautia*, *Faecalibacterium*, and *Prevotella*, representative of the gut microbiota, were determined (Figure S6). It was confirmed that a high level of gut microbes was present in both normal and tumor tissues. In addition, microbiota analysis revealed differences between tissues at the genus level; the level of *Tepidimonas* was around 2.75 times higher in tumor tissues (Figure 1E and Table S2). However, there was no difference in abundance of *Tepidimonas* according to the surgical procedures (Figure S7).

### 2.3. Alterations in Tissue-Specific EV-Derived Microbiomes According to Tumor Progression 

**Figure 2:**
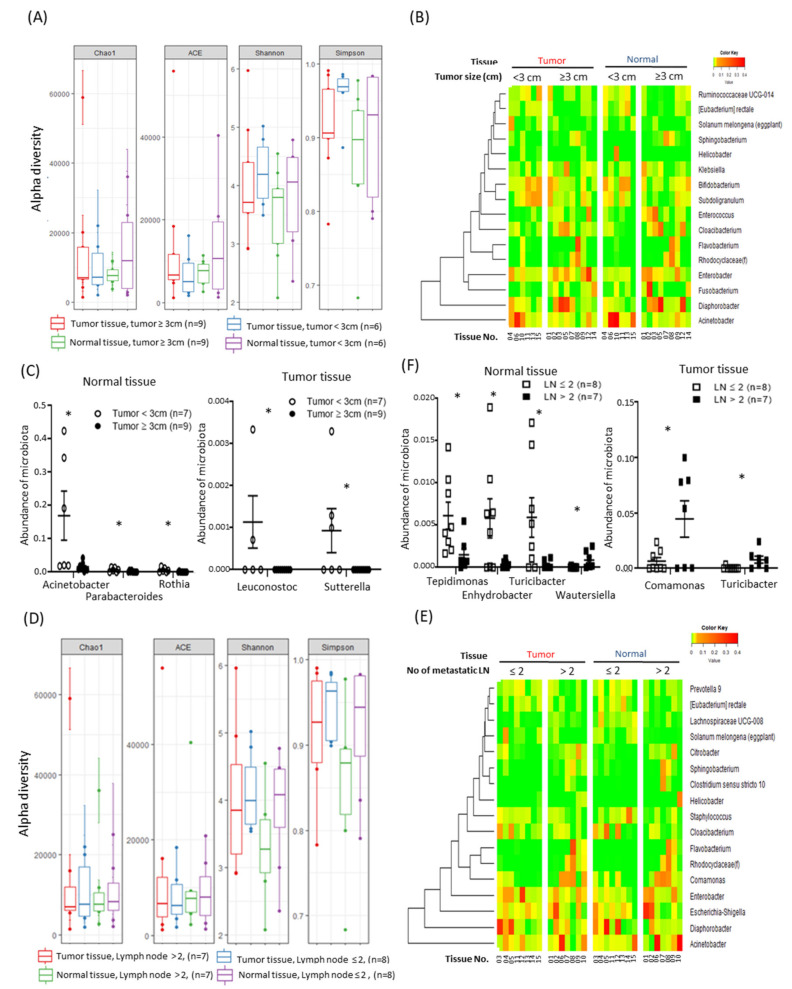
Comparative analysis of the extracellular vesicle (EV)-derived microbiomes according to pathologic findings: (**A**) comparison of alpha diversity indices between tumor and normal tissues according to tumor size, (**B**) clustering of common microbiota in paired normal and tumor tissues according to tumor size, (**C**) specific microbiota taxa with increased abundance in normal and tumor tissues according to tumor size (mean ± SEM, * *p* < 0.05), (**D**) comparison of alpha diversity indices between tumor and normal tissues according to the number of metastatic lymph nodes, (**E**) clustering of common microbiota in paired normal and tumor tissues according to the number of metastatic lymph nodes, and (**F**) Specific microbiota taxa with increased abundance in normal and tumor tissues according to the number of metastatic lymph nodes (mean ± SEM, * *p* < 0.05).

**Figure 3:**
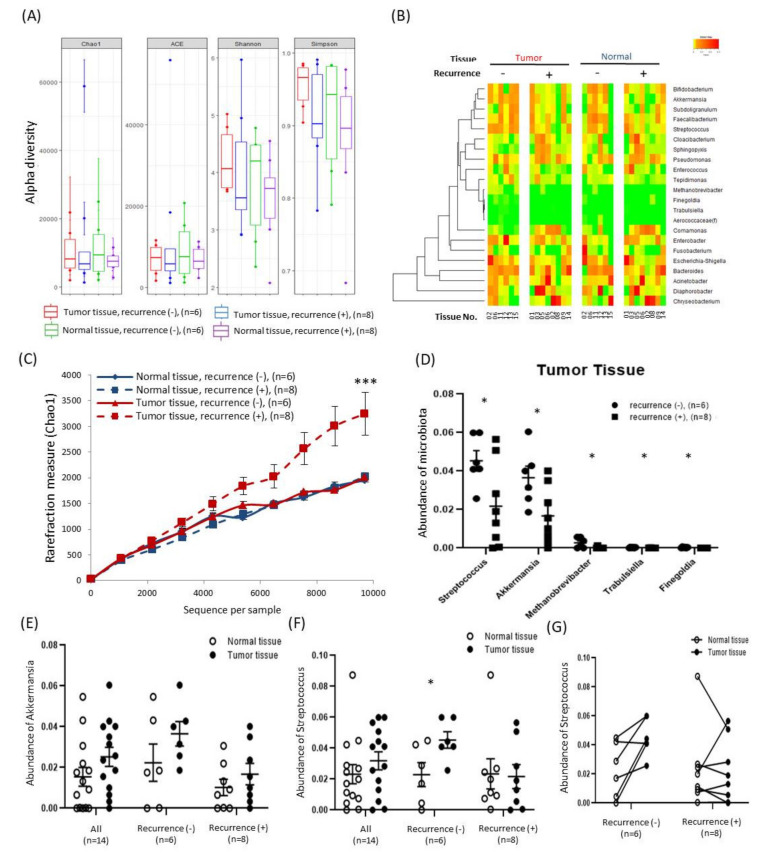
Comparative analysis of the extracellular vesicle (EV)-derived microbiomes according to tumor recurrence: (**A**) comparison of alpha diversity indices between tumor and normal tissues according to the presence of tumor recurrence, (**B**) clustering of common microbiota in paired tumor and normal tissues according to the presence of tumor recurrence, (**C**) alpha diversity rarefaction plots for the EV-derived microbiomes in tumor and normal tissues according to the presence of tumor recurrence (mean ± SEM, *** *p* < 0.001), (**D**) specific microbiota taxa with increased abundance in tumor tissues according to the presence of tumor recurrence (mean ± SEM, * *p* < 0.05), (**E**) comparison of the abundance of *Akkermansia* in tumor and normal tissues according to the presence of tumor recurrence (mean ± SEM, * *p* < 0.05), and (**F**,**G**) comparison of the abundance of Streptococcus in tumor and normal tissues according to the presence of tumor recurrence (mean ± SEM, * *p* < 0.05).

To determine whether the composition of the EV-derived microbiomes present in tissues differ according to tumor progression, additional analyses was performed based on the tumor size, number of metastasized lymph nodes, and presence of tumor recurrence. First, all tissues were divided into two groups based on a tumor size of 3 cm, and the composition and diversity of the microbiomes in tumor and normal tissues were compared in each group. Compositions were compared between groups using the Chao1, ACE, Shannon, and Simpson methods (Figure 2A). Microbiota taxa with a high abundance at the genus level were visualized as heat maps, and hierarchical clustering was performed (Figure 2B). When the tumor size was 3 cm or more, the levels of *Acinetobacter*, *Parabacteroides*, and *Rothia* were lower in normal tissues and the levels of *Leuconostoc* and *Sutterella* were lower in tumor tissues (Figure 2C). Second, all tissues were divided into two groups based on the number of metastasized lymph nodes, and the composition and diversity of the microbiomes in tumor and normal tissues were compared in each group. Compositions were compared between groups using the Chao1, ACE, Shannon, and Simpson methods (Figure 2D). Microbiota taxa with a high abundance at the genus level were displayed as heat maps, and hierarchical clustering was performed (Figure 2E). When the number of metastasized lymph nodes was 2 or more, the levels of *Tepidimonas*, *Enhydrobacter*, *Turicibacter*, and *Wautersiella* were lower in normal tissues and the levels of *Comamonas* and *Turicibacter* were significantly higher in tumor tissues (Figure 2F). Third, patients undergoing surgery were continuously observed and analyzed by dividing them into patients with recurrence (n = 8) and patients without recurrence (*n* = 6). One patient (patient no. 10, Table S1) whose recurrence status was unknown was excluded due to the discontinuation of outpatient observation. The composition and diversity of the microbiomes in tumor and normal tissues were compared, and various alpha diversity techniques and heat maps were used (Figure 3A,B). A comparison of the diversity of each tissue based on the sequences per sample revealed that the diversity was increased significantly in tumor tissues with recurrence (Figure 3C, *p* < 0.001). In addition, at the genus level, the levels of *Streptococcus* and *Akkermansia* were lower in the tumor tissues of patients with recurrence (Figure 3D). In particular, the mean abundance of *Akkermansia* was increased in tumor tissues regardless of recurrence (Figure 3E) and the mean abundance of *Streptococcus* was significantly increased in tumor tissues without recurrence (Figure 3F,G).

### 2.4. Alteration of Oncological Function of Tumor Cell by *Tepidimoas Fonticaldi*

**Figure 4:**
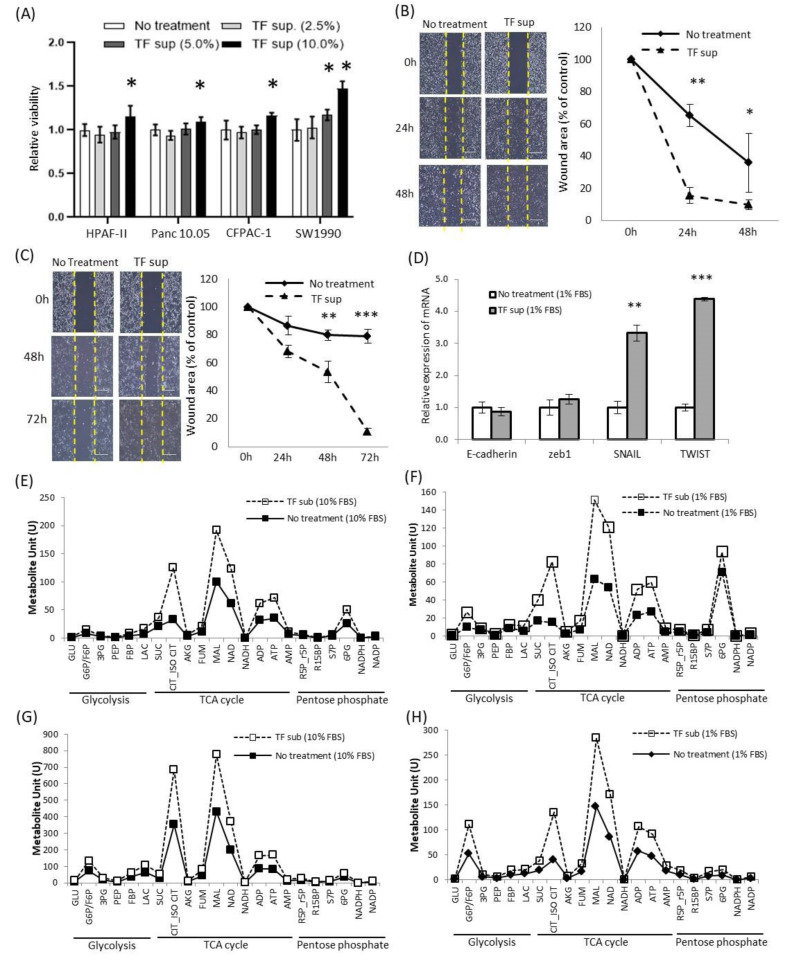
Regulation of oncological function by *Tepidimonas fonticaldi* (TF) in pancreatic cancer cells: (**A**) proliferation of cancer cells in the presence of the supernatant of TF (*n* = 5, mean ± SD, * *p* < 0.05), (**B**) wound healing assay of SW1990 cells in the presence of the supernatant of TF (*n* = 5, mean ± SD, ** *p* < 0.005, and * *p* < 0.05), (**C**) wound healing assay of Panc10.05 cells in the presence of the supernatant of TF (*n* = 5, mean ± SD, ** *p* < 0.005, and *** *p* < 0.001), (**D**) alterations in transcription factors associated with epithelial-mesenchymal transition (EMT) in Panc10.05 cells in the presence of the supernatant of TF (*n* = 4, mean ± SD, ** *p* < 0.005, *** *p* < 0.001), (**E**,**F**) alterations in cancer metabolites in Panc10.05 cells in the presence of the supernatant of TF, and (**G**,**H**) alterations in cancer metabolites in HPAF-II cells in the presence of the supernatant of TF.

Oncological function was evaluated using the supernatant of *Tepidimoas fonticaldi* (TF), one of the species of Tepidimonas with high abundance in tumor tissues (Figure 1E). Cell proliferation was increased according to the ratio of the TF supernatant for HPAF-II, Panc10.05, CFPAC-1, and SW1990 cells (Figure 4A and Figures S8 and S9), and cell migration was increased for SW1990 and Panc10.05 cells (Figure 4B,C). In addition, it was confirmed that the therapeutic efficacy of anticancer drugs could be slightly increased if the supernatants of TF and gemcitabine were simultaneously treated (Figure S10). The mRNA expressions of SNAIL and TWIST and the protein level of SNAIL were increased, and the increase was greater under starvation conditions (fetal bovine serum (FBS) 1%) than under normal conditions (FBS 10%) (Table S3, Figures S11 and S12, and Figure 4D). Furthermore, among the cell’s energy metabolites, metabolites related to the Tricarboxylic Acid TCA cycle, such as citrate, malate (MAL), nicotinamide adenine dinucleotide (NAD), ADP, and ATP, were increased, which were more distinct under starvation conditions (FBS 1%) (Figure 4E–H and Figure S13).

## 3. Discussion

Only a few decades ago, the results of studies of the relationship between *H. pylori* and pancreatic cancer were often not consistent and showed contradictory outcomes [18,19]. However, based on 16s rRNA gene sequencing analysis, it was confirmed that the microbiome could play an important role in the progression of pancreatic cancer and patient survival. *Porphyromonas gingivalis* and *Aggregatibacter actinomycetemcomitans* are oral pathogens associated with an increased risk of pancreatic cancer [11,18], and higher alpha diversity and specific signatures in the tumor microbiome may be associated with the long-term survival of patients with pancreatic cancer [8]. In the case of immune response modulation, the gut microbiome interacts with the immune system and affects cancer progression [20]. An abundant microbiome in pancreatic cancer could induce T-cell anergy via selective Toll-like receptor ligation [7].

We believe that microbiotas in EVs may be one of the pathways for the gut microbiome to play a role in pancreatic cancer. EVs can contain DNA, RNA, proteins, sugars, and lipids and can be produced by any cell type including microbiotas [22,23]. Recently, microbiota-derived EVs have been reported to regulate gut permeability through tight junction regulation, to induce insulin resistance in skeletal muscles, and to be present in patients with lung disease [24,25]. Thus far, there have been no reports of tissue EV-derived microbiotas in pancreatic cancer tissues. In this study, we report for the first time the abundance of EV-derived microbiota in the normal and tumor tissues of patients with pancreatic cancer (Figures S1–S6 and Figure 1). The presence of EV-derived microbiota in the pancreas can be explained in two ways: (1) they may derive from bacteria already residing in the pancreas, and (2) they may be secreted from bacteria in other organs and may reach the pancreas through blood or lymphatic vessels. Further studies are needed to determine which is more likely, and this may change the focus of therapeutic approaches to specific microbiota.

In our study, the alpha diversity based on sequences per sample was significantly higher in tumor tissues (Figure 1A) and the level of *Tepidimonas* was significantly higher in tumor tissues (Figure 1E and Table S2). Studies of *Tepidimonas* in the human body are still limited; *Tepidimonas* has recently been reported to be present in human urine [26]. *Tepidimonas fonticaldi* (TF) is one of the *Tepidimonas* species used in various experiments to examine the effects on pancreatic cancer cell lines [27]. In the presence of the supernatant of TF, proliferation and migration of cancer cells were induced and EMT-related mRNA and TCA cycle-related metabolites were significantly increased (Figure 4 and Figures S11–S13). As these results were obtained using the supernatant of TF and not TF itself, they support the hypothesis that microbiota-derived EVs affect the tumor microenvironment including cancer cells.

Investigation of the differences in tissue-specific EV-derived microbiomes according to tumor progression revealed that the levels of *Acinetobacter*, *Parabacteroides*, and *Rothia* were lower in normal tissues and that the levels of *Leuconostoc* and *Sutterella* were lower in tumor tissues when the tumor size was 3 cm or more (Figure 2C). In addition, when there were 2 or more metastatic lymph nodes, the levels of *Tepidimonas*, *Enhydrobacter*, *Turicibacter*, and *Wautersiella* were lower in normal tissues and the levels of *Comamonas* and *Turicibacter* were higher in tumor tissues (Figure 2F). *Comamonas*, one of the microbiota taxa with a higher abundance in tumor tissues, is a cellulolytic microbe that can affect the metabolism of cancer patients [28,29], which can be used to construct a predictive model of biliary tract cancer [30]. On the other hand, *Turicibacter* is part of the core microbiota of the rat digestive tract [31] and has been reported to be significantly higher in a dog model of multicentric lymphoma [32]. In addition, in a mouse model of colitis-associated colorectal cancer, *Turicibacter* was significantly increased and has been reported to be useful as a treatment target [33].

In addition, analysis of tumor recurrence, which has the greatest effect on the survival of patients with pancreatic cancer, revealed that the alpha diversity of patients with recurrence was significantly increased (Figure 3C). However, the frequency of five microbiota members including *Streptococcus* and *Akkermansia* was rather low (Figure 3D). *Akkermansia muciniphila* (*A. muciniphila*) is a representative species of *Akkermansia* found in human feces, and interest and research on the role of probiotics have been increasing [34,35]. *A. muciniphila* was reported to play a role in the relief of abdominal pain in patients with irritable bowel syndrome [36], and it was more abundant in the feces of lung cancer patients who responded well to immunotherapy [37]. In another study, it significantly reduced the number of tumors in the gastrointestinal tract in a mouse model [38]. A membrane protein purified from *A. muciniphila* reduced colitis-associated tumorigenesis through CD8+ T cell modulation in a mouse model [39] and had beneficial effects on the gut barrier and metabolism through interaction with toll-like receptor 2 [40]. Furthermore, we obtained interesting results in the analysis of Streptococcus by comparing pairs of normal and tumor tissues (Figure 3F,G). In the group without recurrence, the distribution in tumor tissues was higher than that in normal tissues (83.3%, 5/6). However, in the group with recurrence, the distribution in tumor tissues was often similar to or lower than that in normal tissues (87.5%, 7/8). *Streptococcus mitis* (*S*. *mitis*) is a representative microbiota member of *Streptococcus* in the oral microbiome, and various studies related to oral cancer have been conducted [41,42]. The level of *S. mitis* has been reported to be not significantly different or lower in patients with pancreatic cancer compared with healthy individuals [43,44]. In our study, the levels of *Akkermansia* and *Streptococcus* were relatively higher in the tumor tissues of patients without recurrence. It is possible that these microbiota taxa can protect against the development and progression of pancreatic cancer tumors.

## 4. Materials and Methods 

### 4.1. Clinicopathological Characteristics of Patients

This study complied with the Declaration of Helsinki and was reviewed and approved by the Institutional Review Board (IRB) of Asan Medical Center (IRB No. 2018-0710). Patients who underwent surgery for pancreatic cancer were enrolled in this study, and informed consent was obtained. The paired tumor and normal tissues of 15 patients who were pathologically diagnosed as pancreatic ductal adenocarcinoma (PDAC) were delivered from the Bio-resource center (BRC No. 2018-13(167)). Each patient had a follow-up period of at least 4 years, and medical records were retrospectively reviewed for clinicopathological characteristics. Age, sex, preoperative laboratory findings, pathological findings, history of neoadjuvant chemotherapy, and tumor recurrence within 4 years were determined. Preoperative blood tests included WBC (white blood cell), Hb (hemoglobin), Plt (platelet), AST (aspartate aminotransferase), ALT (alanine aminotransferase), ALP (alkaline phosphatase), TP (total protein), Alb (albumin), TB (total bilirubin), BUN (blood urea nitrogen), Crea (creatinine), CA 19–9 (carbohydrate antigen 19–9), and CEA (carcinoembryonic antigen). The surgical procedure was determined according to the tumor location and extension. Pathological findings included tumor size, tumor differentiation, lymphovascular invasion, perineural invasion, and metastatic lymph nodes.

### 4.2. Isolation of EVs and DNA Extraction

Tumor tissues were frozen in liquid nitrogen, and the precooled tissues were ground using a steel bar. After grinding, 500 μL of phosphate-buffered saline (PBS) was added. The mixtures were homogenized by vortexing for 30 s. Each sample was centrifuged at 10,000× *g* for 10 min at 4 °C. The supernatant, comprised of EVs, was passed through a 0.22-μm membrane filter to eliminate foreign particles. The separated bacterial EVs were boiled at 100 °C for 40 min to eliminate the remaining floating particles and waste. They were then centrifuged at 13,000× *g* for 30 min at 4 °C. The supernatants were collected, and protein concentration was determined. Bacterial DNA was extracted from the prepared EVs using a DNA extraction kit (PowerSoil DNA Isolation Kit, MO BIO Laboratories, Solana Beach, CA, USA) following the manufacturer’s instructions. The isolated DNA was quantified using the QIAxpert system (QIAGEN, Hilden, Germany) [45].

### 4.3. PCR Amplification and Sequencing of 16S Rrna Genes 

The prepared bacterial genomic DNA was used for PCR amplification of the V3–V4 hypervariable regions of the 16S ribosomal RNA genes with the primer set of 16S_V3_F (5′-TCGTCGGCAGCGTCAGATGTGTATAAGAGACAGCCTACGGGNGGCWGCAG-3′) and 16S_V4_R (5′-GTCTCGTGGGCTCGGAGATGTGTATAAGAGACAGGACTACHVGGGTATCTAATCC-3′). The PCR products were used for the construction of 16S rDNA gene libraries following the MiSeq System guidelines (Illumina Inc., San Diego, CA, USA). The 16S rRNA gene libraries for each sample were quantified using QIAxpert (QIAGEN, Hilden, Germany), pooled at the equimolar ratio, and used for pyrosequencing with the MiSeq System (Illumina, San Diego, CA, USA) according to the manufacturer’s recommendations. 

### 4.4. Taxonomic Assignment and Analysis of the Bacterial Composition of the Microbiota

Taxonomic assignments were made based on the sequence reads of the 16S rRNA genes as described previously [24]. In brief, paired-end reads that matched the adapter sequences were trimmed by cutadapt (version 1.1.6) [46]. The resulting FASTQ files containing paired-end reads were merged with CASPER and then quality-filtered with Phred (Q) score-based criteria as described by Bokulich [47,48]. Any reads shorter than 350 bp or longer than 550 bp after merging were also discarded. To identify the chimeric sequences, a reference-based chimera was detected by VSEARCH against the SILVA gold database [49]. Subsequently, clustering into operational taxonomic units (OTUs) was conducted using VSEARCH with the de novo clustering algorithm under a 97% sequence similarity. The representative sequences of the OTUs were finally classified using the SILVA 128 database with UCLUST (script on QIIME version 1.9.1) [9].

### 4.5. Comparative Analysis for Microbiome Diversity 

The rarefaction curve of Chao1 was used for alpha diversity metrics using multiple_rarefaction.py and alpha_diversity.py QIIME package. Alpha diversity metrics such as Chao1 (species richness), ACE (species richness), Shannon (>0, higher, more diverse), and Simpson index (0–1, 1 = most simple) were computed to measure the richness and evenness of the communities within groups. All metrics were computed using the “estimate_richness” function in R package phyloeseq ver. 1.22.3 [50]. Beta diversity (distance between samples based on differences in the OTUs present in each sample) was measured by principal component analysis (PCA) based on the Euclidean distance to determine differences in overall metagenomic profiles. Heat maps were used to visualize ordination using selected taxa (average relative abundances were greater than 1%, and fold changes were greater than 2-fold). In addition, hierarchical clustering was applied. 

### 4.6. Culture of Tepidimonas Fonticaldi and Cancer Cells

TF was purchased from the Korean Collection for Type Culture (KCTC, Jeongeup, Korea). Freeze-dried TF in a Pyrex ampule was cultured in 10% tryptic soy agar (TSA) at 37 °C with 5% CO_2_ in an incubator and was stored at 4 °C before use. For in vitro experiments, TF colonies were cultured in 10% tryptic soy broth (TSB) at 37 °C in a shaking incubator, and growth was measured via optical density at 600 nm using a spectrophotometer (M108, CamSpec, Leeds, UK). The supernatant of TF was obtained after centrifugation of the TF culture solution at 4000 rpm for 30 min (4 °C). 

Panc10.05, HPAF-II, CFPAC-1, and SW1990 (ATCC, Manassas, VA, USA) and the Capan2 and SNU213 (KCLB, Seoul, Korea) human pancreatic cell lines were cultured in RPMI-1640 medium (HyClone, Logan, UT, USA) supplemented with 10% *v*/*v* fetal bovine serum (FBS; Hyclone, Logan, UT, USA), 100 U/mL penicillin, and 100 μg/mL streptomycin (Gibco BRL, Grand Island, NY, USA) at 37 °C with 5% CO2 in a humidified incubator 

### 4.7. In Vitro Assay of Cancer Cells

Each assay was independently performed at least three times. For cell proliferation assay, cancer cells were plated at a density of 3 × 103 cells per well in a 96-well culture plate and incubated for 18 h at 37 °C. After adding the supernatant of TF (2.5%, 5%, and 10% *v*/*v*) to each well, the relative cell viability was evaluated by cell counting kit-8 assay (Sigma-Aldrich, St. Louis, MO, USA) after 72 h at 37 °C.

For the wound healing assay, cancer cells were seeded at a density of 1 × 105 cells per well in 35 × 11 mm dishes and incubated for 18 h at 37 °C. After adding the supernatant of TF (10% *v*/*v*) to each dish, the cell layer in each dish was scratched using a plastic pipette tip. The migration of the cells at the edge of the scratch was analyzed at 0, 24, 48, and 72 h when microscopic images of the cells were captured. The images were analyzed by ImageJ software (version 1.8.0_112).

For quantitative polymerase chain reaction (PCR), cancer cells were seeded at a density of 1 × 105 cells per well in 35 × 11 mm dishes and incubated for 18 h at 37 °C. After adding the supernatant of TF (10% *v*/*v*) in 10% or 1% *v*/*v* FBS to each dish, the cells were maintained for 24 h at 37 °C. Total RNA was extracted from the lysed cells using RNeasy Mini Kit (Cat. 74104, QIAGEN, Valencia, CA, USA) according to the manufacturer’s instructions and eluted in 50 µL elution buffer. The concentration and purity of RNA were confirmed using NanoDrop 2000 Spectrophotometer (Thermo Fisher, Waltham, MA, USA), and the RNA was stored at −80 °C until needed for downstream experiments. mRNA expression was analyzed using a LightCycler® 480 instrument (Roche Diagnostics Ltd., Burgess Hill, UK) with SYBR® Green Master Mix. The primer sequences for the genes are listed in Table S3. The real-time qRT-PCR protocol was as follows: 95 °C for 5 min, followed by 40 cycles at 95 °C for 10 s, 58 °C for 20 s, and 72 °C for 10 s. The relative concentrations of PCR products were analyzed using LightCycler® 480 software (Roche Diagnostics Ltd., Burgess Hill, UK). All samples were normalized to 18S [51].

For western blotting, cells were lysed in the radioimmunoprecipitation assay (RIPA) buffer (Sigma-Aldrich, St. Louis, MO, USA) containing protease inhibitors (Thermo Fisher Scientific, Waltham, MA, USA). Total proteins (10–20 µg) were separated by 12% sodium dodecyl sulfate-polyacrylamide gel electrophoresis and detected using primary antibodies targeting the following proteins: beta-actin (sc-47778, 1:10000, Santa Cruz, CA, USA), SNAIL (MA5-14801, 1:1000, Thermo Fisher, Waltham, MA, USA), and horseradish peroxidase-conjugated secondary immunoglobulin antibody (1:20000 dilution, Thermo Fisher, Waltham, MA, USA). Immunocomplexes were visualized with an enhanced chemiluminescence detection kit (Bio-Rad, Hercules, CA, USA).

### 4.8. Metabolite Analysis

Cancer cells were seeded at a density of 1 × 105 cells per well in 35 × 11 mm dishes and incubated for 18 h at 37 °C. After adding the supernatant of TF (10% *v*/*v*) in 10% or 1% *v*/*v* FBS to each dish, the cells were maintained for 24 h at 37 °C. The cells were harvested using 1.4 mL of cold methanol/H_2_O (80/20, *v*/*v*) after rapid and sequential washing with PBS and H_2_O. Then, the cells were lysed by vigorous vortexing, and 100 μL of 5 μM internal standard (13C5-Gln) was added. Metabolites were extracted from the aqueous phase by liquid–liquid extraction after adding chloroform. The aqueous phase was dried using a vacuum centrifuge, and the sample was reconstituted with 50 μL of 50% methanol prior to LC-MS/MS analysis. Metabolites related to energy metabolism were analyzed by LC-MS/MS with the 1290 HPLC system (Agilent Technologies, Palo. Alto, CA, USA), Qtrap 5500 system (ABSciex, Concord, ON, Canada), and a reverse phase column (Synergi Fusion RP 50 × 2 mm; Phenomenex, Torrance, CA, USA). Multiple reaction monitoring (MRM) was performed in the negative ion mode, and the extracted ion chromatogram (EIC) corresponding to the specific transition for each metabolite was used for quantification. The area under the curve of each EIC was normalized to that of the EIC of the internal standard. The peak area ratio of each metabolite to the internal standard was normalized based on the amount of protein. The standard metabolites and internal standard were purchased from Sigma-Aldrich (St. Louis, MO, USA). All solvents including water were purchased from J. T. Baker (Phillipsburg, NJ, USA) [52]. (GLU; glucose, G6P/F6P; glucose-6-phosphate/fructose 6-phosphate, 3PG; 3-phosphoglycerate, PEP; phosphoenol pyruvate, FBP; fructose-1,6-bisphosphate, LAC; lactate, SUC; succinate, CIT/ISO CIT; citrate/isocitrate, AKG; alpha ketoglutarate, FUM; fumarate, MAL; malate, NAD; nicotinamide adenine dinucleotide, NADH; reduced nicotinamide adenine dinucleotide, ADP; adenosine diphosphate, ATP; adenosine triphosphate, AMP; adenosine monophosphate, R5p/r5p; ribulose-5-phosphate/ribose-5-phosphate, R15BP; ribose-1,5-bisphosphate, S7P; sedoheptulose-7-phosphate, 6PG; 6-phosphogluconate, NADPH; reduced nicotinamide adenine dinucleotide phosphate, NADP; nicotinamide adenine dinucleotide phosphate).

### 4.9. Statistics Analysis

Comparisons of the relative abundance of OTUs and alpha diversity between groups were performed with the t-test. Statistical significance was considered if the *p* value was < 0.05. Statistical analyses were performed using R software (ver. 3.6.0) or GraphPad Prism version 8.1 (GraphPad Software, La Jolla, CA, US). Data are expressed as the mean ± standard error (or standard error of the mean) for continuous variables and as frequency for categorical variables.

## 5. Conclusions

In this study, we analyzed EV-derived microbiomes in the paired normal and tumor tissues of patients with pancreatic cancer. In addition, we analyzed changes in the composition and diversity of the microbiomes in both tissues and identified tissue-specific microbiotas according to tumor progression. Based on the results of this study, we plan to conduct studies on the origin of EV-derived microbiomes, interaction with the gut microbiome, and immuno-oncological function within the tumor microenvironment. The results could help clarify the function and role of the microbiome in pancreatic cancer and could contribute to advances in diagnosis and treatment. 

## Figures and Tables

**Table 1:** Clinical and tissue characteristics of enrolled patients. SD; standard deviation, WBC; white blood cell, Hb; hemoglobin, Plt; platelet, AST; aspartate aminotransferase, ALT; alanine aminotransferase, ALP; alkaline phosphatase, TP; total protein, Alb; albumin, TB; total bilirubin, BUN; blood urea nitrogen, Crea; creatinine, CA 19–9; carbohydrate antigen 19–9, CEA; carcinoembryonic antigen, PD; pancreaticoduodenectomy, TPS; total pancreatectomy with splenectomy, DPS; distal pancreatectomy with splenectomy, LN; lymph node, CTx; chemotherapy, n.i.; not identified.

Factors		Values
Age, y	Mean ± SD, (range)	65.0 ± 8.2 (52.0–79.0)
Sex (Female/Male)	N (%)	4/11 (26.7/73.3)
Preoperative laboratory	Mean ± SD, (range)	
WBC (*10^3^/μL)		6.5 ± 1.9 (2.1–9.1 )
Neutrophil (%)		55.6 ± 10.0 (32.3–71.2)
Lymphocyte (%)		34.5 ± 9.4 (21.8–58.0)
Monocyte (%)		6.7 ± 2.0 (2.4–11.8)
Hb (g/dL)		13.6 ± 1.2 (11.7–15.7)
Plt (*10^3^/μL)		210.6 ± 39.0 (144.0–268.0)
AST (IU/L)		27.3 ± 8.9 (20.0–49.0)
ALT (IU/L)		28.7 ± 18.1 (11.0–74.0)
ALP (IU/L))		100.7 ± 48.0 (38.0–211.0)
TP (g/dL)		6.6 ± 0.5 (5.7–7.5)
Alb (g/dL)		3.8 ± 0.5 (2.8–4.6)
TB (mg/dL)		1.1 ± 1.7 (0.2–7.1)
BUN (mg/dL)		12.8 ± 3.1 (9.0–19.0)
Crea (mg/dL)		0.8 ± 0.1 (0.5–1.1)
CA 19-9 (U/mL)		190.3 ± 337.4 (3.4–1290.0)
CEA (U/mL)		2.2 ± 1.1 (0.9–5.5)
Pathologic finding		
Tumor location (head + uncinate/body + tail)	N (%)	5/10 (33.3/66.7)
Operation (PD + TPS/DPS)	N (%)	5/10 (33.3/66.7)
Tumor size (cm)	Mean ± SD, (range)	3.3 ± 1.9 (1.4–9.2)
Tumor differentiation (wel/mod/por)	N (%)	1/13/1 (6.7/86.7/6.7)
Lymphovascular invasion (absent/present)	N (%)	10/5 (66.7/33.3)
Perineural invasion (absent/present)	N (%)	1/14 (6.7/93.3)
metastatic LN (absent/present)	N (%)	5/10 (33.3/66.7)
Neoadjuvant CTx ( non-received/received)	N (%)	14/1 (6.7/93.3)
Recurrence within 4 y (absent/present/n.i)	N (%)	6/8/1 (40.0/53.3/6.7)

## Supplementary Materials

The following are available online at https://www.mdpi.com/2072-6694/12/9/2346/s1, Table S1. Clinicopathological information of enrolled patients. Table S2. Comparative analysis of taxa between normal and tumor tissues. Table S3. PCR primers. ZEB1; zinc finger E-box-binding homeobox 1. Figure S1. Extracellular vesicles (EVs) from the tissues of patients with pancreatic cancer. Figure S2. Beta diversity of tissue EV-derived microbiomes in tumor and normal tissues in 2 dimensions (*n* = 15). Figure S3. Beta diversity of tissue EV-derived microbiomes in tumor and normal tissues in 3 dimensions (*n* = 15). Figure S4. Phylogenetic profiles of EV-derived microbiomes. Figure S5. Microbiome composition profiling in the tissues of patients with pancreatic cancer. Figure S6. The abundance of known important microbiotas in human gut between normal and tumor tissues (*n* = 15). Figure S7. Abundance of *Tepidimonas* by surgical procedures in tumor tissues. Figure S8. Proliferation of cancer cells in the presence of the supernatant of TF in Capan2 and SNU213. Figure S9. Proliferation of cancer cells in the presence of the culture medium of *Tepidimonas fonticaldi* in Panc 10.05 and CFPAC-1 cells. Figure S10. Proliferation of cancer cells in the presence of the supernatant of *Tepidimonas fonticaldi* and Gemcitabine in SW1990 and CFPAC-1 cells. Figure S11. Alterations in the protein of SNAIL with the presence of the supernatant of Tepidimonas fonticaldi in HPAF-II and Panc 10.05 cells. Figure S12. Regulation of transcription factors associated epithelial to mesenchymal transition (EMT) by *Tepidimonas fonticaldi* in Panc 10.05 cells. Figure S13. Alteration of cancer metabolite by the supernatant of *Tepidimonas fonticaldi* in SW1990 cells.
