# Supplementary material for: Diversity in the Extracellular Vesicle-Derived Microbiome of Tissues According to Tumor Progression in Pancreatic Cancer
Source: Cancers (Basel). 2020 Aug 19;12(9):2346. doi: 10.3390/cancers12092346 (PMC7563179; doi:10.3390/cancers12092346)
Supplement: Supplementary file 1 [file cancers-12-02346-s001.pdf]

Supplementary Material

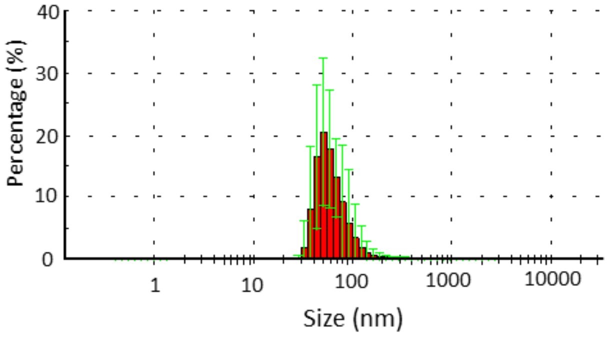

Figure S1. Extracellular vesicles (EVs) from the tissues of patients with pancreatic cancer. Size distribution of EVs obtained from tissues. The average size was 63.42 ± 19.02 nm.

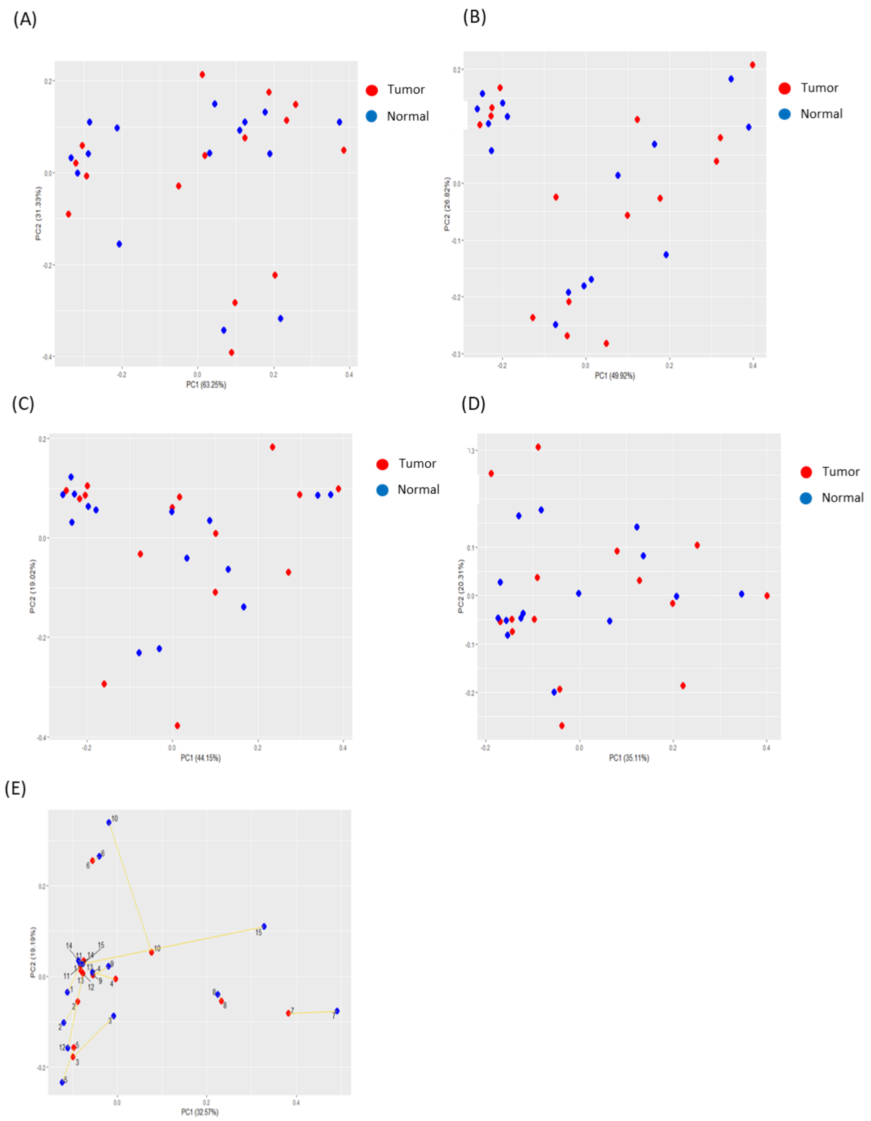

Figure S2. Beta diversity of tissue EV-derived microbiomes in tumor and normal tissues in 2-dimension (n = 15). (**A**) Phylum, (**B**) class, (**C**) order, (**D**) family, and (**E**) genus levels.

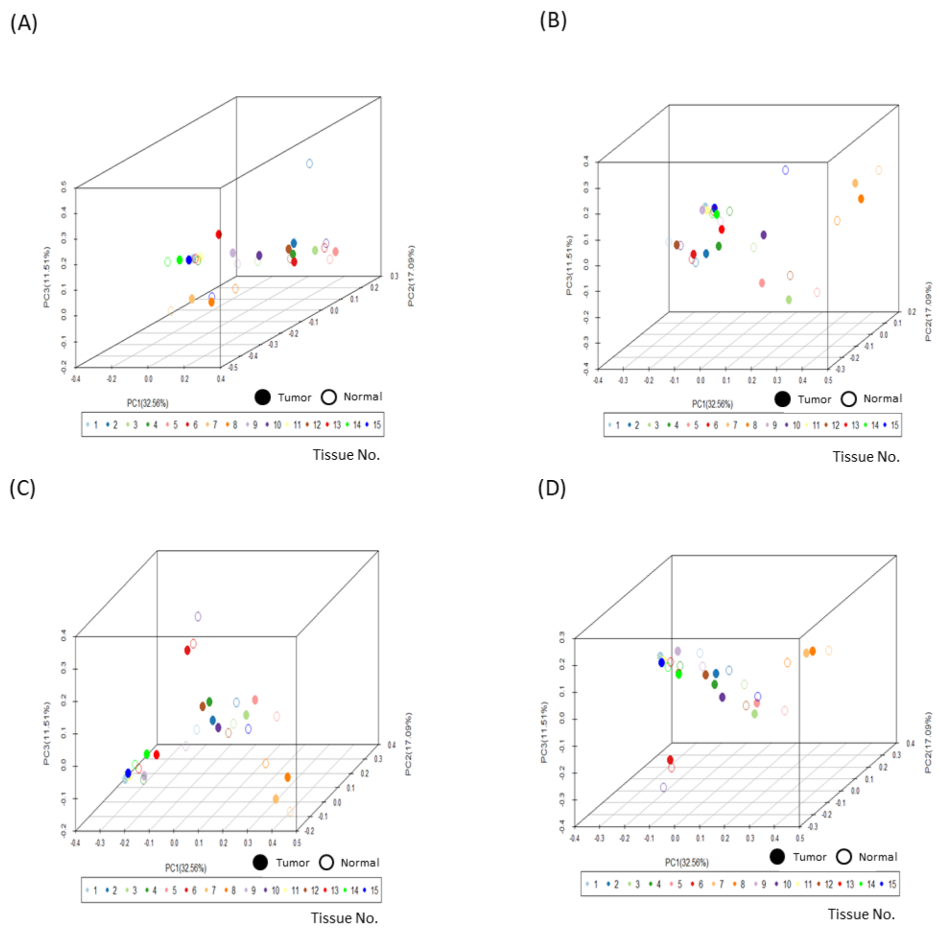

Figure S3. Beta diversity of tissue EV-derived microbiomes in tumor and normal tissues in 3-dimension (n = 15). (**A**) Phylum, (**B**) class, (**C**) order, (**D**) family levels.

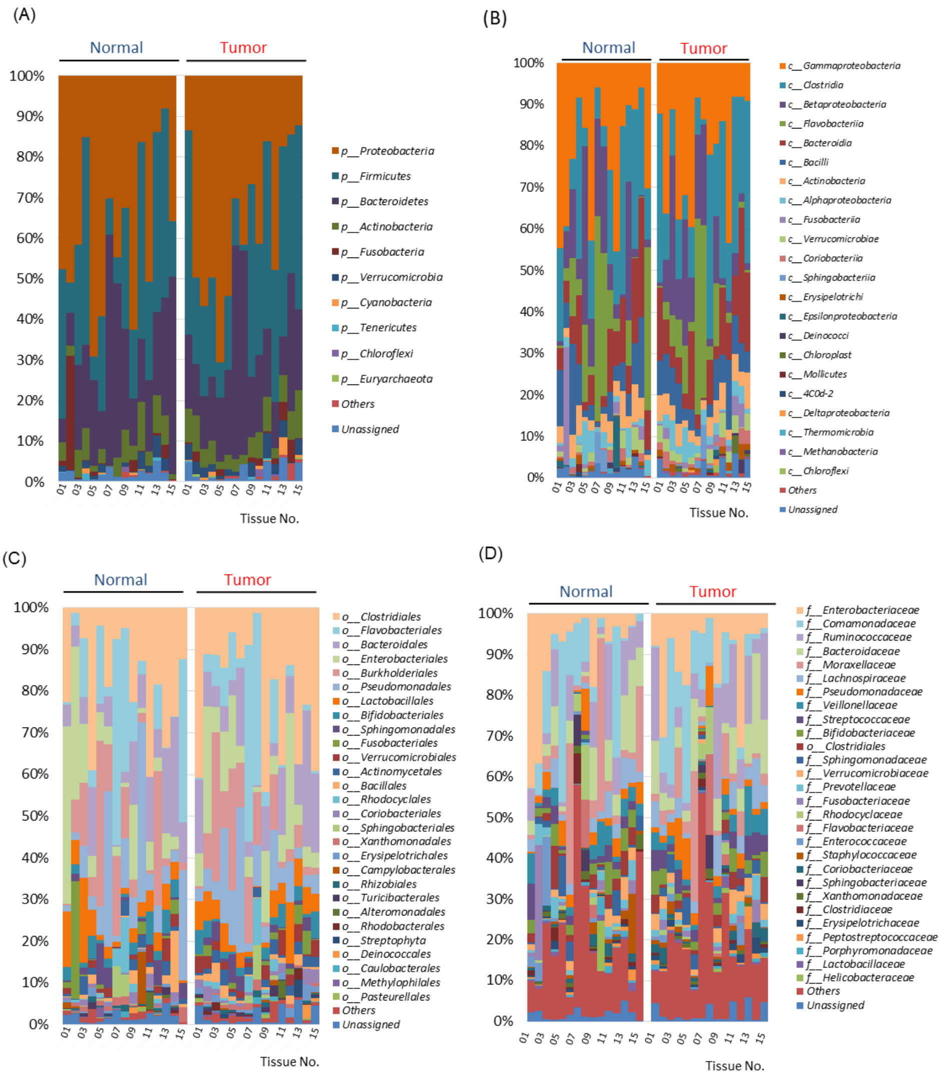

Figure S4. Phylogenetic profiles of EV-derived microbiomes. Common microbiota in paired normal and tumor tissues at the (**A**) phylum, (**B**) class, (**C**) order, and (**D**) family levels.

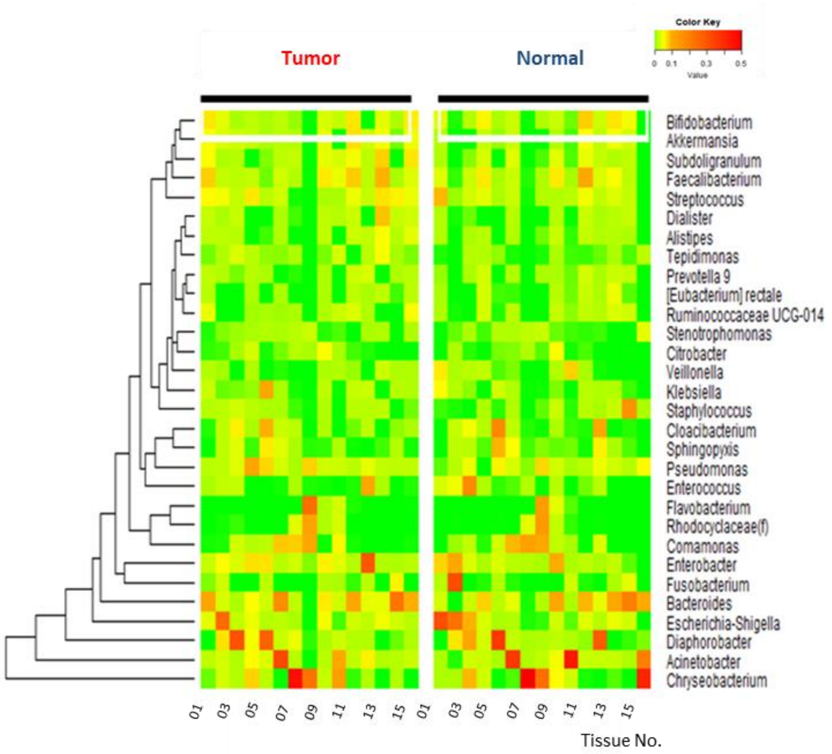

Figure S5. Microbiome composition profiling in the tissues of patients with pancreatic cancer. Clustering of common microbiota in paired normal and tumor tissues (n = 15).

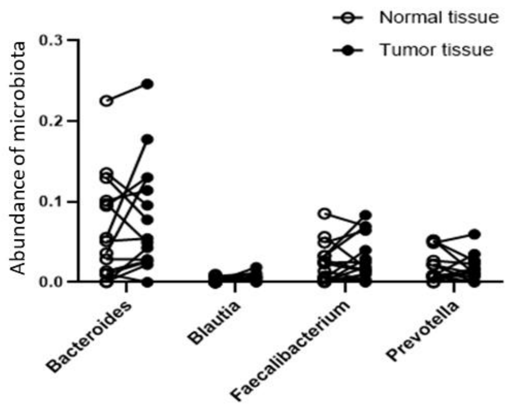

Figure S6. The abundance of known important microbiotas in human gut between normal and tumor tissues (n=15).

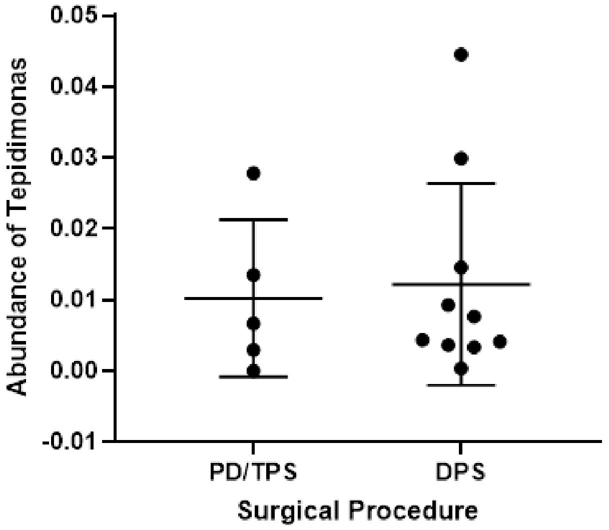

Figure S7. Abundance of *Tepidimonas* by surgical procedures in tumor tissues (PD; pancreaticoduodenectomy, TPS; total pancreatectomy with splenectomy, DPS; distal pancreatectomy with splenectomy, $n = 15$, mean ± SEM).

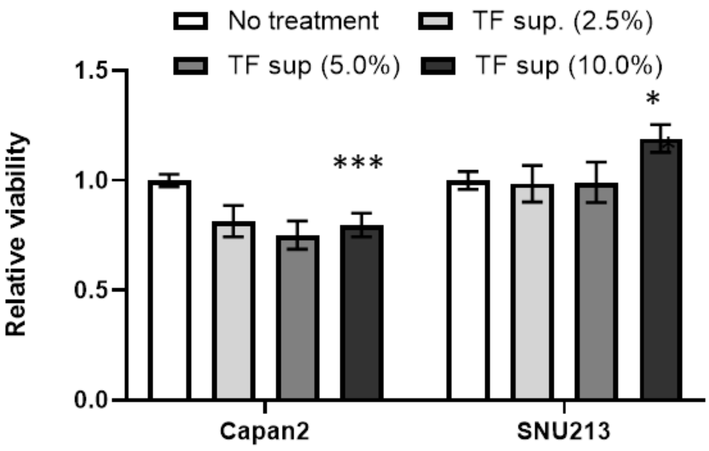

Figure S8. Proliferation of cancer cells in the presence of the supernatant of TF in Capan2 and SNU213 ($n = 5$, mean ± SD, * $p < 0.05$, *** $p < 0.001$).

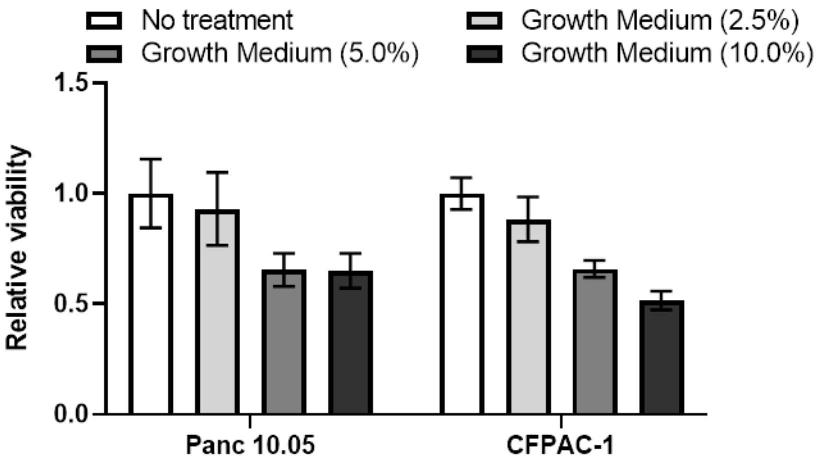

Figure S9. Proliferation of cancer cells in the presence of the culture medium of *Tepidimonas fonticaldi* in Panc 10.05 and CFPAC-1 cell ($n = 5$, mean ± SD).

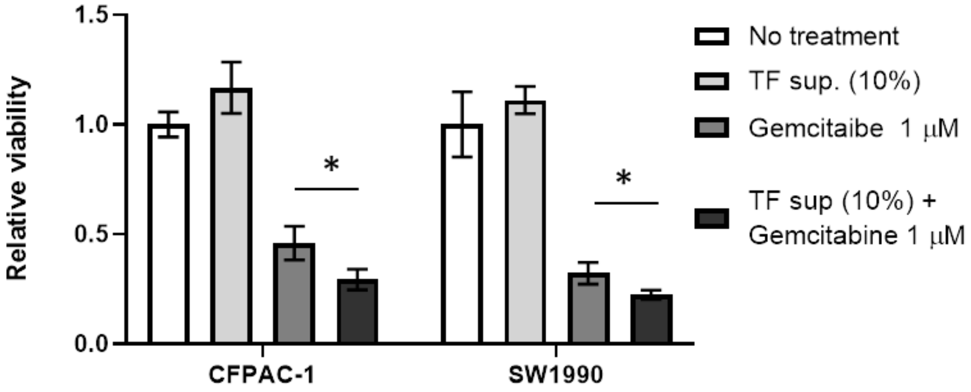

Figure S10. Proliferation of cancer cells in the presence of the the supernatant of *Tepidimonas fonticaldi* and Gemcitabine in SW1990 and CFPAC-1 cell ($n = 5$, mean ± SD, * $p < 0.05$).

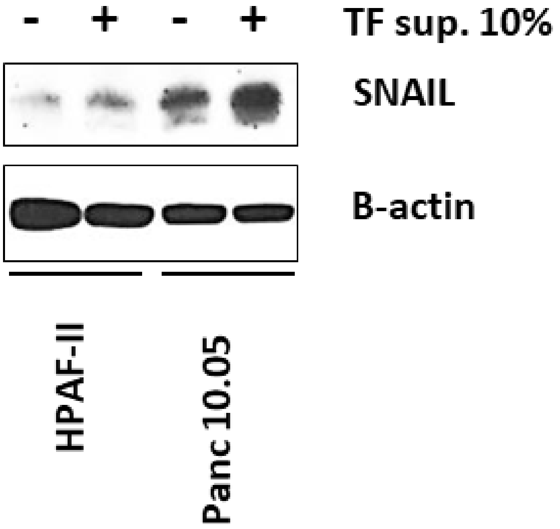

Figure S11. Alterations in protein of SNAIL with the presence of the supernatant of *Tepidimonas fonticaldi* in HPAF-II and Panc 10.05 cell (1% FBS).

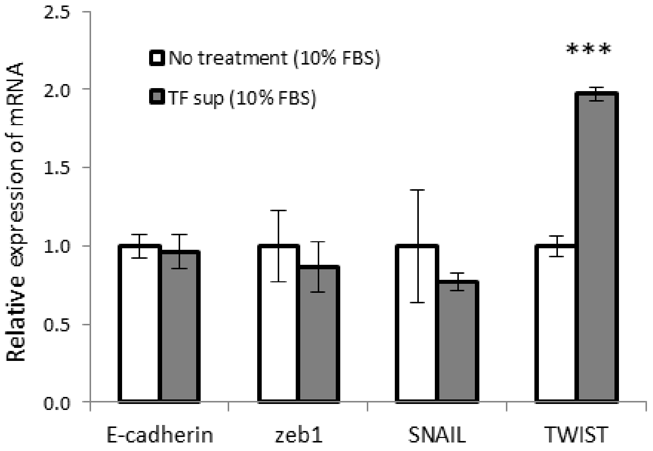

Figure S12. Regulation of transcrpition factors associated epithelial to mesenchymal transition (EMT) by *Tepidimonas fonticaldi* in Panc 10.05 cell (10% FBS, $n = 4$, ***$p < 0.001$).

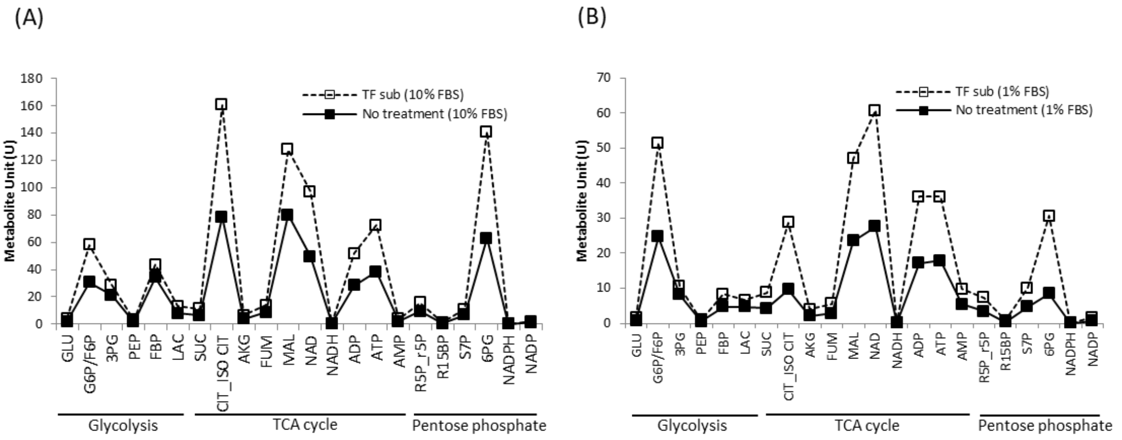

Figure S13. Alterations in cancer metabolites in SW1990 cells in the presence of the supernatant of *Tepidimonas fonticaldi* (TF). Metabolic assay was performed in (**A**) 10% FBS and (**B**) 1% FBS.

Table S1. Clinicopathological information of enrolled patients. WBC; white blood cell, Hb; hemoglobin, Plt; platelet, AST; aspartate aminotransferase, ALT; alanine aminotransferase, ALP; alkaline phosphatase, TP; total protein, Alb; albumin, TB; total bilirubin, BUN; blood urea nitrogen, Crea; creatinine, CA 19-9; carbohydrate antigen 19-9, CEA; carcinoembryonic antigen, DPS; distal pancreatectomy with splenectomy, TPS; total pancreatectomy with splenectomy, PD; pancreaticoduodenectomy, LN; lymph node, n.i.; not identified.

ID	Age (y)	Sex	WBC (*10^3/uL)	Neutrophil (%)	Lymphocyte (%)	Monocyte (%)	Hb (g/dL)	Plt (*10^3/uL)	AST (IU/L)	ALT (IU/L)	ALP (IU/L)	TP (g/dL)	Alb (g/dL)	TB (mg/dL)	BUN (mg/dL)	Crea (mg/dL)
1	52	Male	7.0	49.8	39.5	6.8	12.8	193	20	21	118	6.1	3.7	0.7	9	0.77
2	68	Male	9.1	67.1	24.6	7	14.6	180	25	19	127	6.4	4	0.7	16	0.76
3	64	Male	4.7	71.2	21.8	5.5	13	162	23	12	56	6.7	4.2	0.8	11	0.81
4	60	Male	6.5	45.8	45.6	4.9	14	179	20	22	62	6.8	4.3	1.6	11	0.71
5	69	Female	5.9	61.4	30.1	7.8	14.6	194	21	17	121	6.7	4.1	0.4	10	0.48
6	57	Male	6.5	60.5	32	5.6	15.2	264	33	36	91	7.5	4.3	0.9	13	0.98
7	74	Male	8.8	61.1	25.9	11.8	11.8	216	49	74	174	6.1	2.8	7.1	13.6	0.95
8	52	Male	9.1	58.1	31.5	7.7	14.9	268	26	21	93	7.5	4.6	0.7	14	1.06
9	61	Male	7.6	60.5	29.1	5.6	13.4	216	30	52	76	6.9	4.3	0.4	10	0.74
10	79	Male	5.6	40.9	44.5	7.4	13.5	217	43	55	211	5.7	3.4	1.4	10	0.71
11	71	Male	8.4	59.2	31.3	7.6	13.7	183	22	29	38	6.6	3.6	0.5	10	0.72
12	73	Female	4.5	56.1	35	6.9	12.1	224	33	22	130	6.5	3.6	0.2	12	0.77
13	66	Female	6.0	57.2	31.3	7.5	13.3	258	21	15	58	6.4	3.6	0.3	18	0.7

14	71	Female	2.1	32.3	58	2.4	11.7	261	21	11	52	6.4	3.3	0.2	19	0.73
15	58	Male	6.0	52.6	37.5	5.7	15.7	144	22	25	103	7.1	3.9	0.4	15	0.89

Table S1. (Continued). Clinicopathological information of enrolled patients. WBC; white blood cell, Hb; hemoglobin, Plt; platelet, AST; aspartate aminotransferase, ALT; alanine aminotransferase, ALP; alkaline phosphatase, TP; total protein, Alb; albumin, TB; total bilirubin, BUN; blood urea nitrogen, Crea; creatinine, CA 19-9; carbohydrate antigen 19-9, CEA; carcinoembryonic antigen, DPS; distal pancreatectomy with splenectomy, TPS; total pancreatectomy with splenectomy, PD; pancreaticoduodenectomy, LN; lymph node, n.i.; not identified.

ID	CA19-9 (U/mL)	CEA (ng/mL)	Operation type	Tumor location	Tumor Size (cm)	Tumor differentiation	Lymphovascular Invasion	Perineural Invasion	Metastatic LN	No. of metastatic LN	Neoadjuvant chemotherapy	Recurrence within 4 y
1	8.5	2	TPS	head	9.2	mod	present	present	present	4	none	present
2	1290	2.1	DPS	body	3.1	mod	absent	present	present	10	none	absent
3	393	1.7	DPS	body	5.1	mod	absent	present	present	1	none	present
4	22	2.6	DPS	tail	2.2	mod	absent	present	absent	0	none	absent
5	17.7	3	DPS	body	4.0	mod	absent	present	absent	0	none	present
6	254	2.9	DPS	body	2.6	wel	present	present	present	6	none	present
7	3.4	2.6	PD	head	3.0	mod	absent	present	present	4	none	present
8	9.7	1.3	PD	neck	3.0	mod	absent	present	present	4	none	present
9	57.9	5.5	DPS	body	3.2	mod	absent	present	present	3	none	present
10	244.9	1.8	PD	head	1.8	2	present	present	present	6	none	n.i
11	428.9	1.7	DPS	tail	1.4	mod	present	present	present	2	none	absent
12	18.4	1.2	TPS	head	3.1	mod	absent	present	absent	0	recienved	absent
13	11.2	0.88	DPS	body	1.7	mod	absent	absent	absent	0	none	absent
14	40.4	1.6	DPS	tail	3.5	mod	present	present	present	2	none	present
15	53.9	1.9	DPS	body	2.4	mod	absent	present	absent	0	none	absent

Table S2. Comparative analysis of taxa between normal and tumor tissues.

Taxon	Normal Tissue		Tumor Tissue		t test
	Mean	SD	Mean	SD	*p*-Value
Bacteria;p_Bacteroidetes;c_Bacteroidia;o_Bacteroidales;f_Bacteroidaceae;g_Bacteroides	0.066	0.064	0.081	0.068	0.232
Bacteria;p_Bacteroidetes;c_Flavobacteriia;o_Flavobacteriales;f_[Weeksellaceae];g_Chryseobacterium	0.088	0.156	0.065	0.124	0.440
Bacteria;p_Proteobacteria;c_Gammaproteobacteria;o_Enterobacteriales;f_Enterobacteriaceae;g_	0.065	0.094	0.059	0.069	0.808
Bacteria;p_Firmicutes;c_Clostridia;o_Clostridiales;f_Ruminococcaceae;g_	0.041	0.035	0.056	0.052	0.258
Bacteria;p_Proteobacteria;c_Betaproteobacteria;o_Burkholderiales;f_Comamonadaceae;g_	0.060	0.109	0.055	0.090	0.806
Bacteria;p_Proteobacteria;c_Gammaproteobacteria;o_Pseudomonadales;f_Moraxellaceae;g_Acinetobacter	0.077	0.134	0.047	0.080	0.223
Bacteria;p_Proteobacteria;c_Gammaproteobacteria;o_Pseudomonadales;f_Pseudomonadaceae;g_Pseudomonas	0.029	0.028	0.037	0.034	0.375
Bacteria;p_Firmicutes;c_Clostridia;o_Clostridiales;f_Ruminococcaceae;g_Faecalibacterium	0.024	0.024	0.031	0.028	0.307
Bacteria;p_Firmicutes;c_Bacilli;o_Lactobacillales;f_Streptococcaceae;g_Streptococcus	0.023	0.023	0.030	0.022	0.215
Bacteria;p_Proteobacteria;c_Betaproteobacteria;o_Burkholderiales;f_Comamonadaceae;g_Comamonas	0.028	0.041	0.024	0.035	0.519
Bacteria;p_Actinobacteria;c_Actinobacteria;o_Bifidobacteriales;f_Bifidobacteriaceae;g_Bifidobacterium	0.026	0.023	0.024	0.022	0.744
Bacteria;p_Verrucomicrobia;c_Verrucomicrobiae;o_Verrucomicrobiales;f_Verrucomicrobiaceae;g_Akkermansia	0.015	0.017	0.024	0.018	0.077
Bacteria;p_Proteobacteria;c_Gammaproteobacteria;o_Enterobacteriales;f_Enterobacteriaceae;g_Citrobacter	0.014	0.026	0.021	0.025	0.325
Unassigned;Other;Other;Other;Other;Other	0.018	0.014	0.020	0.021	0.682
Bacteria;p_Firmicutes;c_Clostridia;o_Clostridiales;f_;g_	0.025	0.028	0.020	0.019	0.561
Bacteria;p_Proteobacteria;c_Gammaproteobacteria;o_Enterobacteriales;f_Enterobacteriaceae;Other	0.016	0.026	0.020	0.027	0.631
Bacteria;p_Firmicutes;c_Clostridia;o_Clostridiales;f_Veillonellaceae;g_Dialister	0.008	0.010	0.019	0.025	0.115
Bacteria;p_Firmicutes;c_Clostridia;o_Clostridiales;f_Lachnospiraceae;g_	0.016	0.017	0.018	0.014	0.746
Bacteria;p_Bacteroidetes;c_Flavobacteriia;o_Flavobacteriales;f_[Weeksellaceae];g_Cloacibacterium	0.025	0.048	0.016	0.025	0.278
Bacteria;p_Bacteroidetes;c_Flavobacteriia;o_Flavobacteriales;f_Flavobacteriaceae;g_Flavobacterium	0.010	0.031	0.016	0.051	0.392
Bacteria;p_Bacteroidetes;c_Bacteroidia;o_Bacteroidales;f_Prevotellaceae;g_Prevotella	0.019	0.018	0.015	0.016	0.303
Bacteria;p_Proteobacteria;c_Betaproteobacteria;o_Rhodocyclales;f_Rhodocyclaceae;g_	0.014	0.035	0.012	0.029	0.625
Bacteria;p_Proteobacteria;c_Betaproteobacteria;o_Burkholderiales;f_Comamonadaceae;g_Tepidimonas	0.004	0.004	0.011	0.013	*0.035*
Bacteria;p_Firmicutes;c_Clostridia;o_Clostridiales;f_Ruminococcaceae;g_Ruminococcus	0.005	0.009	0.010	0.011	0.231
Bacteria;p_Fusobacteria;c_Fusobacteriia;o_Fusobacteriales;f_Fusobacteriaceae;g_Fusobacterium	0.025	0.072	0.009	0.015	0.371

Table S3. PCR primers. ZEB1; zinc finger E-box-binding homeobox 1.

Gene		Sequence (5′->3′)	Annealing temperature (°C)	RT-PCR product length (bp)
18s	Forward	CGGCTACCACATCCAAGGAA	60	187
	Reverse	GCTGGAATTACCGCGGCT		
E-cadherin	Forward	TTGCACCGGTCGACAAAGGAC	63	231
	Reverse	TGGATTCCAGAAACGGAGGCC		
ZEB1	Forward	TGCACTGAGTGTGGAAAAGC	58	237
	Reverse	TGGTGATGCTGAAAGAGACG		
Snail	Forward	GGCTCCTTCGTCCTTCTCCT	60	124
	Reverse	CTGGAGATCCTTGGCCTCAG		
Twist	Forward	TGCGGAAGATCATCCCCACG	63	137
	Reverse	GCTGCAGCTTGCCATCTTGGA		
